# DXA reference values and anthropometric screening for visceral obesity in Western Australian adults

**DOI:** 10.1038/s41598-020-73631-x

**Published:** 2020-10-30

**Authors:** Jonathan M. D. Staynor, Marc K. Smith, Cyril J. Donnelly, Amar El Sallam, Timothy R. Ackland

**Affiliations:** 1 School of Human Sciences M408 (Exercise and Sport Science), The University of Western Australia, 35 Stirling Hwy, Crawley, WA 6009 Australia; 2 Body Composition Technologies, Pty Ltd, South Perth, WA Australia; 3 My Fiziq, PLC, South Perth, WA Australia; 4 Rehabilitation Research Institute of Singapore (RRIS), Nanyang Technological University, Singapore, Singapore; 5 School of Computer Science and Software Engineering, The University of Western Australia, Crawley, WA Australia

**Keywords:** Anatomy, Risk factors, Predictive markers

## Abstract

Limited reference values exist for visceral adipose tissue (VAT) mass measured by DXA. The objectives of this study were to provide reference values for DXA-derived VAT mass and compare the association with anthropometry measures. The study cohort comprised 677 men and 738 women aged 18–65 years from Western Australia. Whole-body scans using a GE Lunar iDXA and anthropometry measures were collected. Reference percentile data were stratified by sex and age. Correlation analysis compared DXA-derived and anthropometry variables. Specificity, sensitivity, and Youden’s Index were used to evaluate the ability of anthropometric thresholds to predict individuals with high VAT. In men, waist circumference (WC), waist-hip ratio, and waist-height ratio (WHtR) had ‘high’ correlations with VAT mass. In women, only WHtR was ‘highly’ correlated with VAT mass. Overweight thresholds for WC, along with a body mass index of 25.0 kg/m^2^ in women, had the highest combination of sensitivity and specificity when using anthropometry measures to identify individuals with high VAT mass. We provide the first reference data sets for DXA-derived VAT mass among Western Australians. Excessive VAT mass may be identified in men using the overweight WC threshold and in women using both the overweight BMI and WC thresholds.

## Introduction

Visceral adipose tissue (VAT) is a fat depot located within the abdominal cavity, in close proximity to the internal organs^1^. VAT is a significant predictor of incident cardiovascular disease, type 2 diabetes, cancer, and mortality^2,3^. Compared with subcutaneous fat, VAT secretes free fatty acids and adipocytokines (inflammatory markers), with direct access to the liver through the portal vein^1^. Within the liver, these biomarkers promote insulin resistance, liver fat accumulation, and hypertension, as well as other risk factors indicative of cardiovascular disease and type 2 diabetes^1,4^. The predictive capabilities and mechanistic pathway of VAT with non-communicable diseases and mortality highlight the importance of reliably measuring VAT to identify ‘at-risk’ individuals in need of targeted intervention.

VAT measurement has been mostly limited to magnetic resonance imaging (MRI), computed tomography (CT) and dual energy X-ray absorptiometry (DXA) scans. MRI and CT methods have limited availability for general population screening due to the high cost and extensive medical use of these devices, exposure to radiation (for CT), and need for manual image segmentation of the VAT region by trained clinicians or testers. Developments in DXA analysis software provide the ability to indirectly estimate VAT mass and volume within the android region, with significant savings in both data processing time and imaging costs. Kaul et al.^5^, showed that DXA-derived VAT volume was highly correlated (R^2^ = 0.96) with CT outputs, with a small mean difference of 56 cm^3^. DXA also exposes individuals to less radiation than CT (0.96 µSv compared with 3,100 µSv for an abdominal CT). Additionally, DXA machines can scan and output several additional body composition measures, including percent body fat (%BF) and android fat mass, with minimal addition to imaging, segmentation and analysis times.

Most published reference data are limited to anthropometric estimates, such as body mass index (BMI), waist circumference (WC), waist-hip ratio (WHR), and waist-height ratio (WHtR), which are surrogate measures of adiposity and body fat distribution. Due to their ease of measurement, anthropometry variables are preferentially used as screening tools for health risk. Expert consensus^6^ and findings from a meta-analysis^7^, show that anthropometric waist measures have greater discriminatory power than BMI to predict adverse health risk. For example, among 20–30 years old Polish participants, WC correlated better with VAT, rather than WHR or BMI. Further research is needed to investigate the use of anthropometric cut-offs for VAT mass screening among cohorts with a broader range of age and ethnicity characteristics.

Normative DXA body composition data are useful for identifying ‘high-risk’ individuals within a population. Specifically, measures of VAT have been shown to considerably improve the prediction of cardiovascular disease, cancer, diabetes and mortality incidence beyond that of BMI and WC estimates^2,8^. DXA-derived whole-body and regional adiposity measures have been published previously. For example, Imboden et al.^9^, and Kelly et al.^10^, have both published reference standards for samples of primarily White adults, however, VAT mass was not included.

When surveying the literature, there is a paucity of research reporting normative VAT measures for different population cohorts across the globe. Of the published literature available, Miazgowski et al.^11^, provided reference values for VAT derived from 421 Polish adults aged 20–30 years. Normative VAT data have also been published for 649 adults aged 18–75 years from the United States^12^ and 3219 adults aged 18–83 years from the United Kingdom^13^. Data from Swainson et al.^13^, were reported in tabular form, as point estimates for the 1st, 2.5th, 50th, 97.5th, and 99th percentiles. As there are limited published VAT mass values, further research is needed to provide normative VAT mass values among other populations around the world.

The aims of this research were to: (1) provide normative data of VAT mass and other body composition variables measured by DXA among a heterogeneous Western Australian population; (2) assess the strength of association between commonly used anthropometric measures and DXA adiposity measures for representing VAT mass; and (3) test the sensitivity and specificity of commonly used anthropometric measures for identifying individuals with excessive VAT mass (90th percentile or greater). We hypothesize that ‘high’ or ‘very high’ correlations will be found between VAT mass and other DXA-derived measures of adiposity, and that there will be ‘moderate’ correlations between VAT mass and traditional anthropometric measures of adiposity^14^. Furthermore, we hypothesize that WC, WHR, WHtR and %BF would better screen individuals with high VAT than BMI.

## Results

All DXA-derived body composition estimates were repeatable with intra- and inter-tester intra-class correlations values of 0.999, in a sub-sample of 32 men and women. Baseline characteristics of the cohort stratified by sex and age are shown in Table 1. Participant ages ranged from 18.0 to 65.4 years and BMI ranged from 14.2 to 48.8 kg/m^2^. Several datapoints were found to be five standard deviations greater than the mean, however, upon further investigation the participants were not deemed to be outliers. Some participants (n = 33) returned a measurement of zero grams of VAT mass and were kept in the analysis.

**Table 1:** Anthropometric and DXA-derived body composition variables by sex and age group.

	Age (years)					
	All (18–65)	18–24	25–34	35–44	45–54	55–65
**Females**						
N	738	163	208	123	147	97
Height (cm)	165.9 ± 6.5	166.9 ± 6.9	166.3 ± 6.5	166.6 ± 6.1	165.8 ± 6.1	162.9 ± 6.2*
Weight (kg)	63.5 (57.4–71.0)	60.5 (54.7–66.3)	63.8 (58.5–71.0)*	66.0 (59.3–72.3)	63.5 (57.9–72.9)	64.2 (57.4–71.2)
Body mass index (kg/m^2^)	23.0 (21.1–25.6)	21.8 (19.9–23.2)	23.3 (21.3–25.6)*	23.3 (21.6–26.4)	23.2 (21.4–26.2)	24.0 (21.7–27.0)
Waist circumference (cm)	72.2 (67.9–79.3)	68.6 (64.8–72.0)	71.8 (67.8–77.0)*	74.7 (68.8–80.5)	74.8 (69.7–82.2)	76.4 (70.4–84.4)
Waist-hip ratio	0.75 ± 0.06	0.73 ± 0.05	0.74 ± 0.05	0.75 ± 0.05	0.76 ± 0.06	0.77 ± 0.06
Waist-height ratio	0.44 (0.41–0.48)	0.41 (0.39–0.44)	0.43 (0.41–0.47)*	0.45 (0.41–0.48)	0.45 (0.42–0.49)	0.47 (0.43–0.51)
Visceral adipose tissue (kg)	0.17 (0.07–0.36)	0.07 (0.02–0.14)	0.16 (0.07–0.30)*	0.22 (0.10–0.45)	0.24 (0.13–0.50)	0.43 (0.17–0.78)*
Abdominal fat (kg)	1.10 (0.69–1.77)	0.80 (0.58–1.18)	1.11 (0.71–1.68)*	1.20 (0.69–1.93)	1.29 (0.77–2.03)	1.54 (1.00–2.34)
Android-gynoid fat ratio	0.32 ± 0.13	0.26 ± 0.09	0.30 ± 0.11*	0.32 ± 0.13	0.34 ± 0.13	0.40 ± 0.14*
Percentage body fat (%)	30.3 ± 7.9	27.7 ± 6.6	29.8 ± 7.6*	30.2 ± 8.6	31.3 ± 8.0	34.3 ± 7.9*
Fat mass index (kg/m^2^)	6.8 (5.2–8.7)	5.9 (4.9–7.0)	6.8 (5.2–8.6)*	6.9 (5.1–8.8)	7.2 (5.6–9.4)	8.2 (6.1–10.1)
**Males**						
N	677	153	212	146	85	81
Height (cm)	179.3 ± 7.2	179.8 ± 7.9	180.5 ± 6.7	178.3 ± 7.6	179.0 ± 5.6	177.5 ± 7.3
Weight (kg)	82.5 (74.7–91.9)	76.7 (69.8–85.5)	83.0 (76.5–90.2)*	83.3 (75.6–93.2)	86.8 (79.1–96.7)	84.2 (74.6–95.7)
Body mass index (kg/m^2^)	25.8 (23.5–28.0)	24.1 (22.2–25.9)	25.8 (23.4–27.7)*	26.0 (24.1–28.6)	27.4 (25.5–29.6)	26.5 (24.7–29.2)
Waist circumference (cm)	83.7 (78.2–92.0)	77.9 (74.8–82.8)	83.0 (78.2–88.77)*	85.2 (80.6–92.9)*	93.3 (85.1–99.8)*	92.2 (85.2–100.1)
Waist-hip ratio	0.86 ± 0.07	0.81 ± 0.04	0.84 ± 0.05*	0.87 ± 0.06*	0.91 ± 0.07*	0.92 ± 0.07
Waist-height ratio	0.47 (0.44–0.51)	0.44 (0.41–0.46)	0.46 (0.43–0.50)*	0.48 (0.45–0.51)*	0.52 (0.48–0.56)*	0.52 (0.48–0.55)
Visceral adipose tissue (kg)	0.43 (0.24–1.00)	0.21 (0.112–0.35)	0.37 (0.22–0.62)*	0.51 (0.31–1.09)*	1.14 (0.54–1.85)*	1.23 (0.66–1.83)
Abdominal fat (kg)	1.30 (0.74–2.20)	0.79 (0.51–1.16)	1.20 (0.73–1.88)*	1.47 (0.91–2.26)	2.25 (1.42–3.31)*	2.19 (1.62–3.09)
Android-gynoid fat ratio	0.51 ± 0.20	0.38 ± 0.12	0.46 ± 0.15*	0.54 ± 0.18*	0.67 ± 0.19*	0.69 ± 0.21
Percentage body fat (%)	22.3 ± 7.6	18.7 ± 5.6	21.2 ± 7.2*	23.0 ± 8.4	26.4 ± 7.0*	26.3 ± 6.8
Fat mass index (kg/m^2^)	5.4 (3.9–7.5)	4.2 (3.3–5.3)	5.1 (3.8–6.7)*	5.8 (4.2–7.9)	7.0 (5.1–9.1)*	7.3 (5.6–8.8)

Normally distributed variables represented by mean ± standard deviation. Non-normally distributed variables represented with median (25th percentile–75th percentile). A bold * denotes *p* < 0.0063 versus preceding age group.

**Figure 1:**
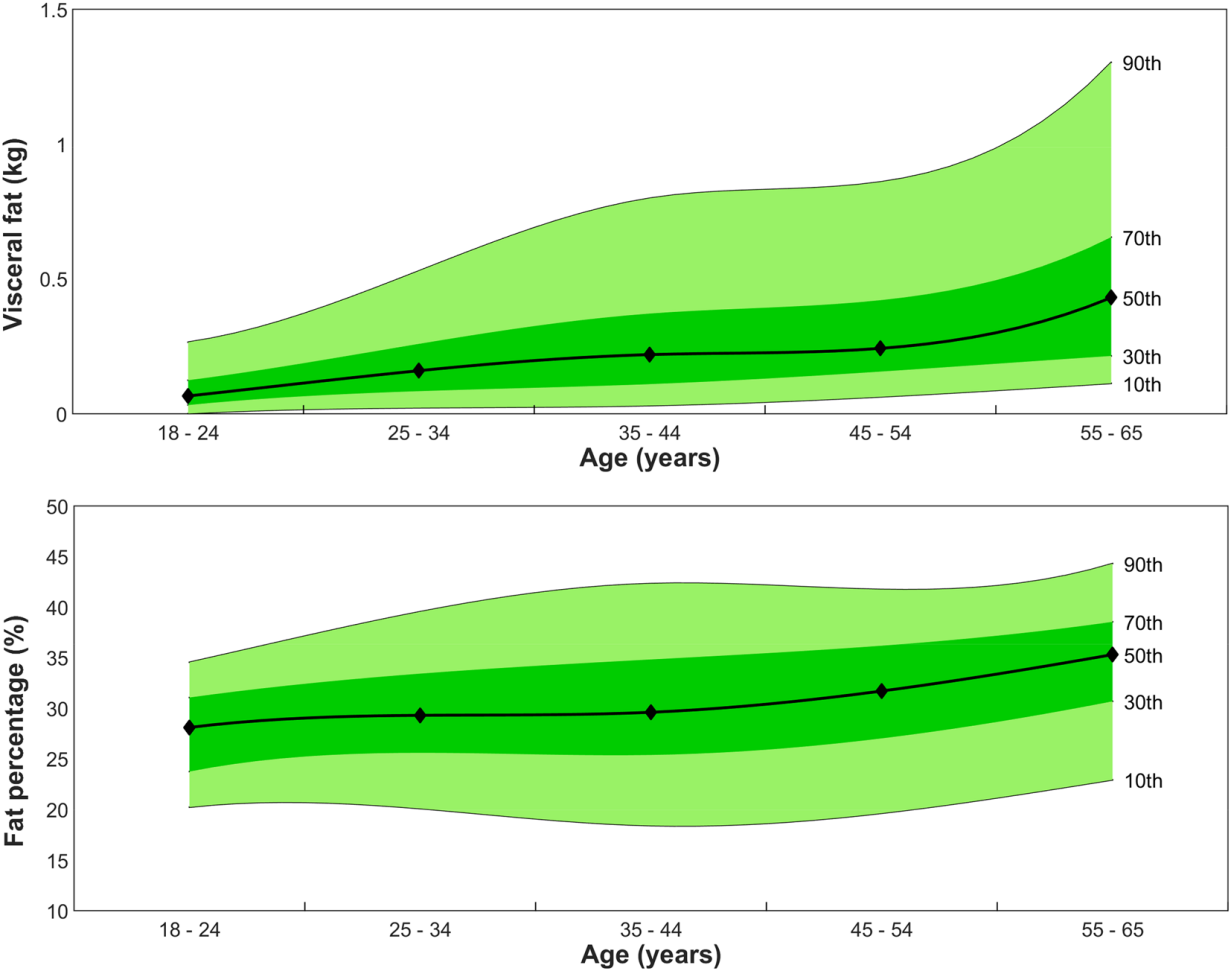
Female normative distributions across age for VAT mass (kg) (top) and %BF (bottom). Solid lines, from top–bottom, represent the 90th, 70th, 50th, 30th and 10th percentiles, respectively.

**Figure 2:**
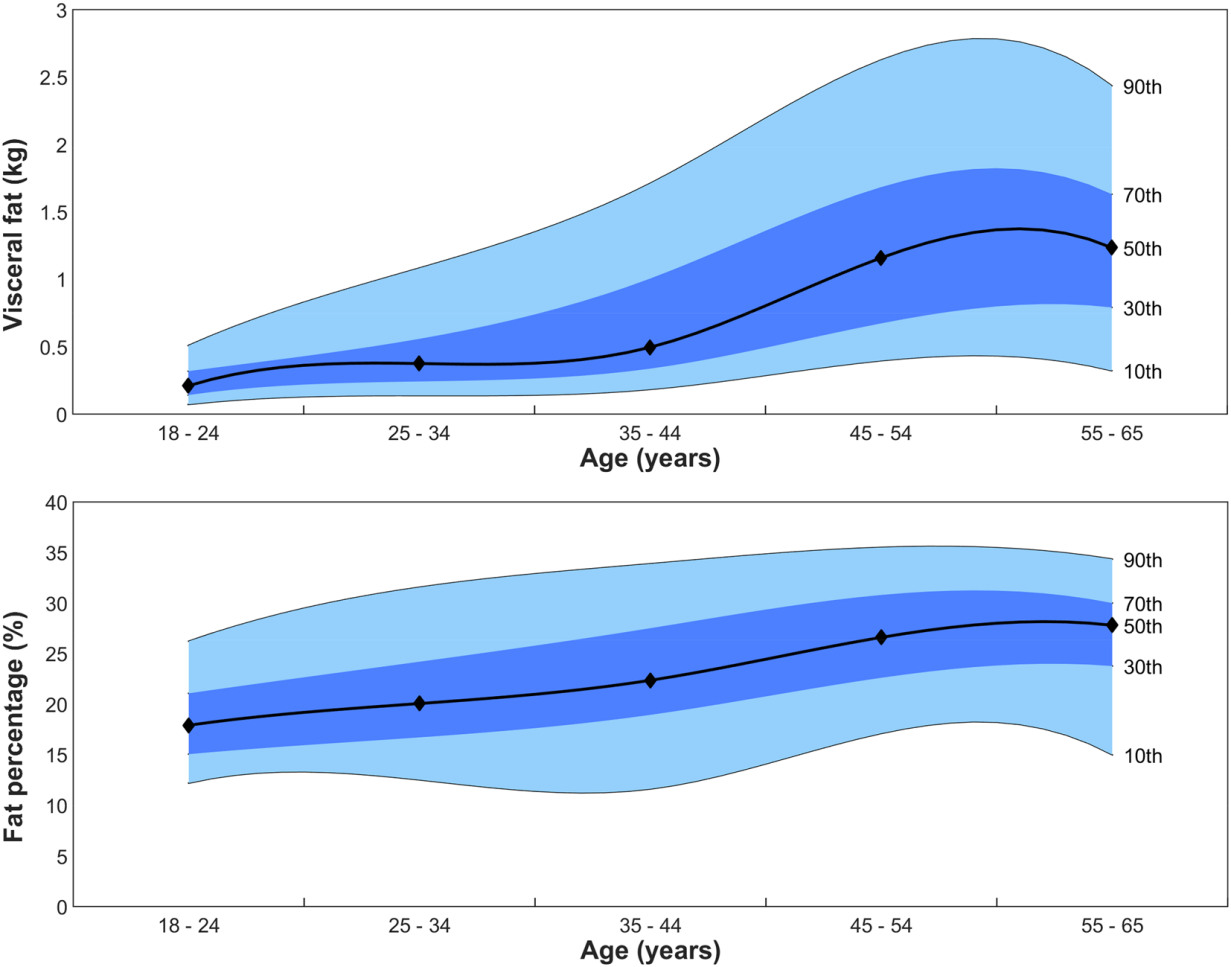
Male normative distributions across age for VAT mass (kg) (top) and %BF (bottom). Solid lines, from top–bottom, represent the 90th, 70th, 50th, 30th and 10th percentiles, respectively.

Significant differences in anthropometry and body composition (p < 0.0063) were recorded between the female 25–34 years and 35–44 years age groups and between the 45–54 years and 55–64 years age groups (Table 1). For men, differences between the current and succeeding age group category were observed for the 25–34, 35–44 and 45–55 age groups. DXA-derived measures of adiposity are presented graphically using spline-interpolation to depict five percentile curves across the age categories. These graphs, for VAT mass and %BF (Figs. 1 and 2), as well as android fat mass, AGR, and FMI (see supplementary file, S1), are presented for clinical comparison with individual results.

‘Moderate’, ‘high’ or ‘very high’ correlation coefficients (p < 0.05) were observed between DXA-derived variables (Table 2). ‘High’ correlations with VAT mass were found for android fat mass and AGR among men. For both sexes, ‘very high’ correlations were observed between android fat mass with %BF and FMI, as well as between %BF and FMI. Correlation coefficients (p < 0.05) ranging from ‘low’ to ‘high’ were observed between VAT mass and anthropometric variables for women (Table 2). Among men, WC, WHR, and WHtR displayed ‘high’ correlations with VAT mass, whereas for females, only WHtR was ‘highly’ correlated with VAT mass.

**Table 2:** Inter-correlations across DXA-derived variables and surrogate anthropometric measures of adiposity.

Sex	Variable	Visceral adipose tissue mass	Android fat mass	Percent body fat	Fat mass index	Android-Gynoid ratio
Female (n = 738)	Visceral adipose tissue mass	1.00				
	Android fat mass	0.78	1.00			
	Percent body fat	0.72	**0.93**	1.00		
	Fat mass index	0.74	**0.96**	**0.96**	1.00	
	Android-gynoid ratio	0.76	0.89	0.78	0.77	1.00
	Body mass index	0.67	0.79	0.69	0.85	0.60
	Waist circumference	0.69	0.79	0.64	0.75	0.69
	Waist-hip ratio	0.46	0.38	0.26	0.29	0.61
	Waist-height ratio	0.72	0.77	0.66	0.77	0.72
Male (n = 677)	Visceral adipose tissue mass	1.00				
	Android fat mass	0.85	1.00			
	Percent body fat	0.75	**0.95**	1.00		
	Fat mass index	0.78	**0.98**	**0.98**	1.00	
	Android-gynoid ratio	0.85	0.89	0.77	0.81	1.00
	Body mass index	0.66	0.74	0.61	0.76	0.61
	Waist circumference	0.80	0.85	0.72	0.81	0.76
	Waist-hip ratio	0.74	0.70	0.65	0.64	0.81
	Waist-height ratio	0.79	0.84	0.74	0.83	0.80

Pearson’s correlations were computed when both variables had normally distributed data, otherwise Spearman’s rho was computed. All correlations were significant (p < 0.00008). ‘Very high’ correlations (r > 0.9) are shown in bold.

**Figure 3:**
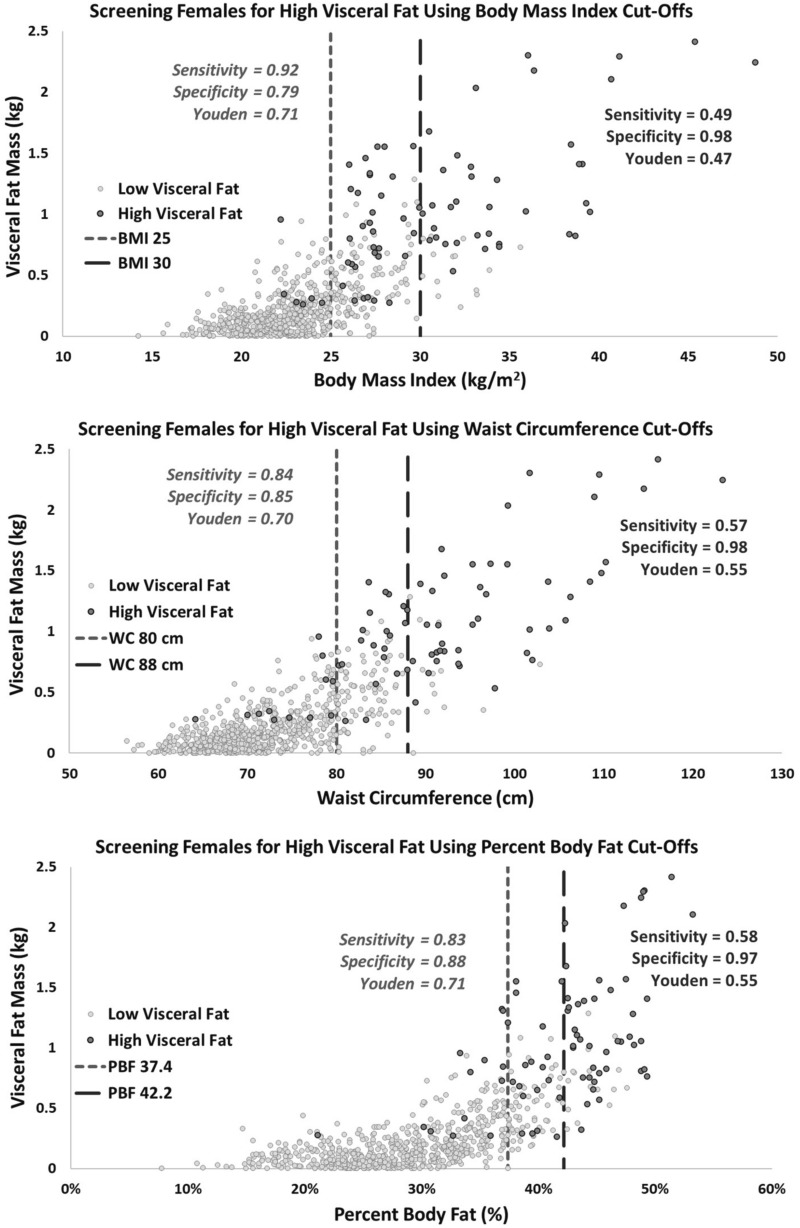
Sensitivity, specificity and Youden’s Index results for identifying women with ‘high VAT’ mass (≥ 90th percentile for age and sex stratified VAT mass) using BMI (top), WC (middle) and %BF (bottom) cut-offs for overweight and obesity.

**Figure 4:**
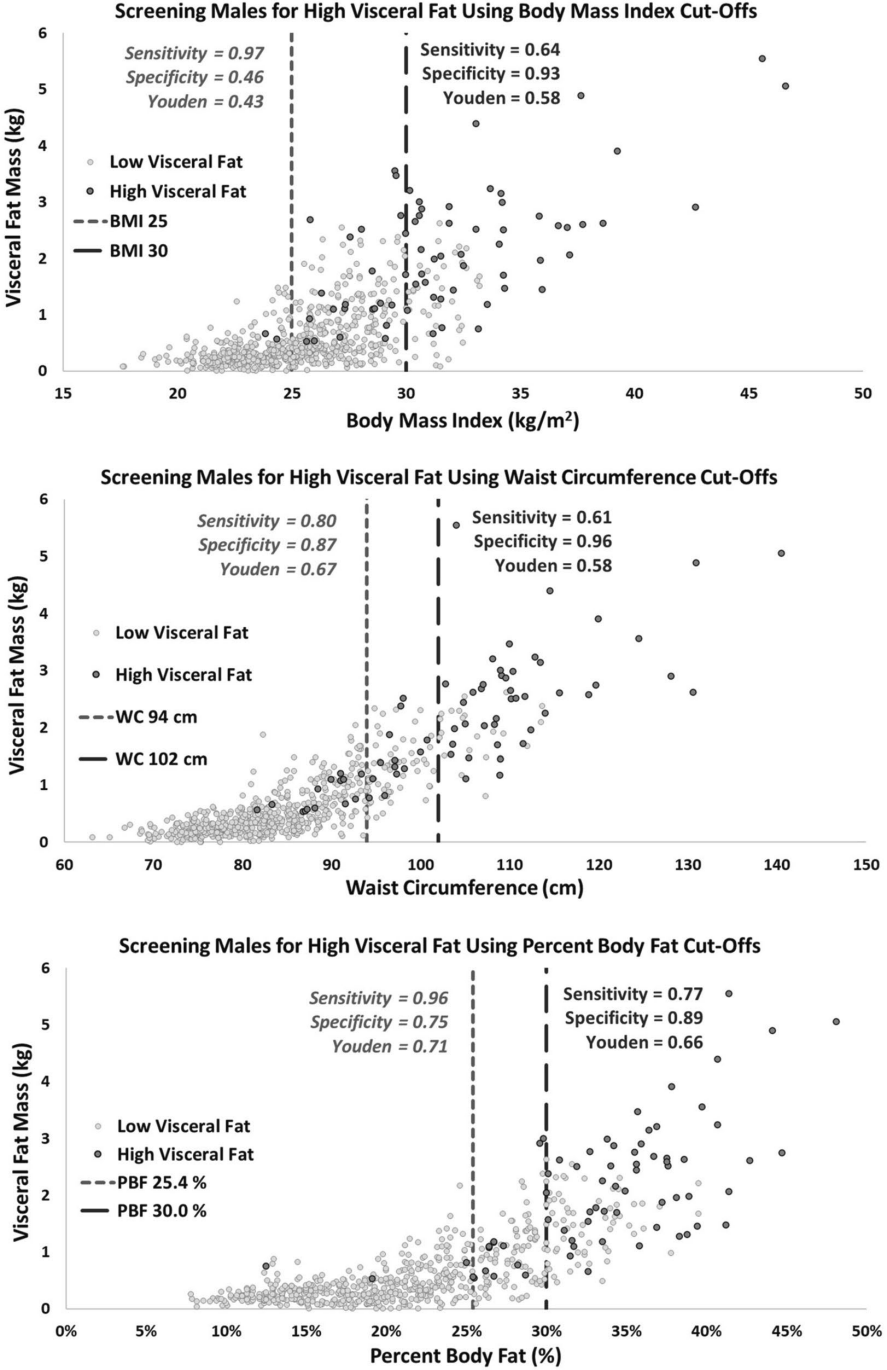
Sensitivity, specificity and Youden’s Index results for identifying men with ‘high VAT’ mass (≥ 90th percentile for age and sex stratified VAT mass) using BMI (top), WC (middle) and %BF (bottom) cut-offs for overweight and obesity.

Scatterplots of anthropometric measures and VAT mass, along with the corresponding sensitivity, specificity, and Youden’s Index for detecting individuals with high VAT mass (90th percentile or greater) are presented in Figs. 3 and 4 for BMI, WC, and %BF, and in supplementary material (S2) for WHR and WHtR. For women, an overweight BMI, %BF, and WC, provided the best screening representation of high VAT individuals, with a Youden’s Index of 0.71, 0.71, and 0.70, respectively. For men, the largest Youden’s Index results of 0.71, 0.67, and 0.67 for screening high VAT individuals were observed by using the overweight cut-offs for %BF, WC, and WHtR, respectively.

## Discussion

The study had two main purposes; the first being to provide sex-specific normative reference values for VAT mass and other DXA-derived adiposity measures for Western Australian adults aged 18–65 years. The normative figures have potential benefit in clinical and wellness settings where individuals are scanned by DXA machines. The second purpose of this study was to identify DXA and anthropometric measurements most capable of screening individuals with high levels of VAT mass. For both men and women, WC and WHtR were more highly correlated with VAT mass when compared to BMI. This also extended to WHR among the male population. In both men and women, the overweight WC cut-off (94 and 80 cm, respectively) was better at screening for high VAT mass than the obese WC cut-off (102 and 88 cm, respectively). When using the obese WC cut-offs^15^, 39% and 43% of men and women with high VAT were not detected. This trend was also observed using BMI^15^, with the overweight cut-offs performing better than the obese cut-off which missed 51% of women and 36% of men with high VAT mass. In Western Australia, it may be prudent to use the lower WC cut-off value (92 cm) for men, and both the lower WC (80 cm) and lower BMI (25.0 kg/m^2^) cut-offs for women to screen for individual’s carrying high levels of VAT.

Age and sex-specific normative DXA adiposity data have been previously reported for populations in the United States^9,10,12^, Poland^11^, and the United Kindgom^13^. Compared with male reference data from the United States^9,10^, the current Western Australian cohort tended to be 2–7% lower in %BF across the age spectrum. The female Western Australian cohort’s %BF was up to 11% lower when compared with the United States cohort. Similar differences were seen for the FMI measures; the Western Australian sample had lower median FMI (approximately 1–4 kg/m^2^) across the age spectrum when compared to the United States sample. Differences in whole-body adiposity may be partially explained by the higher obesity rates seen in the United States^16^. However, these differences, especially in women, suggest that the current cohort may have higher muscle mass and/or less fat mass. Compared to reference data reported by Imboden et al.^9^, the sex and age stratified AGR for the current study appear slightly smaller, suggesting less predisposition to store fat in the android compared to the gynoid region in this Australian cohort.

Age stratified VAT mass for men were similar to those reported in the United States^12^, the United Kingdom^13^, and a 20–30 year old Polish cohort^11^. Australian men may store more VAT for a given %BF; reflected by a lower %BF than their United States counterparts^12^. Female VAT mass values in the current cohort are consistently lower than other normative VAT mass ranges reported previously from different regions globally^11–13^, reinforcing the possibility that women in this study are less predisposed to non-communicable disease. Prospective research measuring body composition, anthropometric data and long-term health outcomes are needed to confirm this assertion. The normative whole-body and region-specific adiposity values provided in this study are the first, that we are aware of, to provide reference values for an Australian population and may be useful in clinical settings for identifying people with high levels of adipose tissue who may be at increased risk of obesity-related diseases.

Differences in anthropometry (BMI, WC, and WHtR) and body composition (VAT mass, abdominal fat mass, AGR, %BF, and FMI) were observed between women aged 18–24 and 25–34 years. After 25–34 years, most anthropometry and DXA variables plateau with no further changes between age groups until women reach 45–54 and 55–65 years of age. Previous cohort studies tended to observe more consistent increases in adiposity with age^9,10,12,13^. It is possible that, in the current sample, total adiposity and abdominal fat distribution remain stable in women, until menopause at around 50 years^17^. Among men there are more consistent differences in anthropometry and body composition, with WC, WHR, WHtR, VAT, and AGR all greater than the preceding age groups for the 25–34, 35–44, and 45–54 year old age groups, despite minimal differences in weight and BMI across these ages. This result suggests that adiposity, particularly VAT mass, increases with age among Western Australian men until it plateaus between 45–54 and 55–65 years. Given the higher VAT mass and increasing trend at an earlier age, Australian males may be more at-risk to non-communicable disease and premature mortality when compared with their female counterparts. While confounding factors, such as physical activity level and other lifestyle factors were not measured or analyzed in this study, the higher levels of VAT at an earlier age are a potential concern to Western Australian men’s long-term risk of non-communicable disease.

As hypothesized, DXA-derived body composition measures had higher correlations with VAT mass than surrogate anthropometric measures. For both men and women, %BF, FMI, android fat mass, and AGR were ‘highly’ correlated with VAT mass. The higher correlations for android fat mass with VAT mass among men may be explained by their propensity to store fat in this area of the body^18^.

Anthropometric measures of BMI and WC were ‘moderately’ correlated with VAT mass in women; with WHtR being ‘highly’ correlated. WHR had the lowest correlation with VAT mass in women (r = 0.46) and had ‘low’ correlations with all of the body composition outputs except AGR which was ‘moderately’ correlated. WHR is a commonly recommended measure to screen for at-risk individuals in clinical settings^6^, and the current results suggest that WC and WHtR may be more appropriate for representing underlying body composition in Western Australian women. In men WC, WHR, and WHtR were all ‘highly’ correlated with VAT mass, outperforming BMI (r = 0.66). This is reflected in Ashwell and colleague’s 2012 systematic review and meta-analysis^7^ of over 300,000 individuals from diverse ethnic backgrounds; where WHtR and WC were found to be superior predictors of diabetes, dyslipidemia, hyper-tension, and cardio-vascular disease risk compared with BMI.

The overweight %BF cut-off for men and women and the overweight BMI cut-off in women were the best predictors of age stratified high (90th percentile or greater) VAT mass (Youden’s Index = 0.71), outperforming the higher obesity cut-offs. Less importance is placed on the overweight thresholds, but there may be merit in referring individuals who exceed these cut-offs for further body composition and health risk assessments. The lower, overweight WC cut-off also performed well when identifying men and women with high VAT (Youden’s Index = 0.67 and 0.70, respectively). This is perhaps logical, given that increased WC is associated with larger amounts of VAT mass^6^. WHtR had no added benefit to WC for both men and women. Together these results suggest that the lower overweight cut-offs for %BF, WC, and BMI should be used to screen for individuals with high VAT and, therefore at risk of associated health conditions.

For men, both BMI cut-offs and the WHR threshold were relatively poor surrogate measures for identifying individuals with high VAT mass. A WHR of 0.85 was particularly poor in women, missing 76% of women with high VAT mass. Based on these results, mass screening of WC among both sexes, along with BMI for women, provides a simple solution to identify individuals with high VAT mass. This supports a recent consensus statement, recommending the routine measurement of WC in clinical practice to improve patient health management^6^. %BF also represents a promising surrogate measure for identifying individuals with high VAT mass, however, the ability to accurately measure %BF in clinical and at-home settings is currently limited.

We acknowledge several limitations. First, the normative sample presented contains several ethnic groups which, other than Whites, are in relatively small proportions. As such, the normative values do not represent specific ethnicities, but rather act as a global overview of expected measurements among Western Australians. Future work should aim to develop ethnicity specific normative data, that may better elucidate differences in body shape and composition, as well as predisposition to chronic disease. A second limitation may be due to recruitment bias as participants were recruited from the Sport Science Department and associated health clinics. These participants are potentially more health conscious than the average Australian, which may in part explain why median whole-body adiposity values were lower than those previously reported^9,10^. A strength of this study is that it is the first to provide normative DXA values, especially for VAT mass, among a large Western Australian cohort. The normative distributions presented may be useful to screen and facilitate early intervention for people with body composition phenotypes associated with an increased risk of chronic disease.

A third limitation is the smoothing of the anthropometry and DXA percentiles across age groups. The data are inherently cross-sectional and smoothing alters the percentile lines. We believe that our approach, which includes greater resolution of percentile lines, will provide clarity for clinicians, allowing them to visually identify an individual’s percentile, avoiding the requirement for calculation. The use of cross-sectional data, however, may limit the interpretation of individual changes in body composition over time.

DXA, especially in young lean females, often returns a value of zero VAT mass. In our study, 33 participants recorded zero grams of VAT, which is unlikely to be accurate. DXA estimates VAT mass from a measure of subcutaneous adipose tissue within the android region, which may explain the occurrence of zero grams of VAT. We chose to leave these VAT values and participants within the study, to better represent the range of values that can be expected when using iDXA to measure VAT mass.

## Conclusion

The use of DXA as a medical imaging device is becoming a frequent methodology in clinical health and wellness settings. We provided the first available reference data sets for DXA-derived VAT mass for a Western Australian cohort. These reference data will allow clinicians and health professionals to compare individual results against expected population values and infer potential health risks. We also provide recommendations for using ‘overweight’ WC thresholds for men and women (94 and 80 cm, respectively), and the ‘overweight’ BMI threshold (25 kg/m^2^) in women, to screen for excessive VAT mass. Use of these thresholds in clinical settings has the potential to highlight people who should be referred for additional body composition and health assessments, as well as given appropriate advice to reduce long-term disease risk.

## Methods

### Participants

All participants were Western Australian residents from the Perth metropolitan area and were recruited from the University of Western Australia’s Sport Science Department and associated health clinics between July 2017 and April 2019. The inclusion criteria required participants to be aged between 18 and 65 years with no apparent physical abnormalities. 1,512 participants were recruited for the study. Participants were excluded if they had a physical disability that prevented an accurate measurement of their anthropometry or body composition (n = 2). All participants with duplicate, incomplete, errored, or missing data were also removed from analysis (n = 71). Visual assessment was performed on all iDXA scans and 24 participants were removed due to poor scan quality, incorrect positioning of the participant, or if significant body segment interaction occurred. Analysis was completed on 1415 participants (677 males and 738 females), comprising 1163 who self-reported as White and 252 who self-reported as another ethnicity (e.g., Asian, Hispanic, Middle Eastern etc.). The University of Western Australia Ethics Committee approved the study protocol (RA/4/1/6084) and all the participants gave their informed written consent. All methods were performed in accordance with the relevant guidelines and regulations.

### Anthropometric measurements

Standing height and body mass, as well as WC and hip circumference (HC) were recorded with participants wearing minimal clothing. Anthropometric measurements were undertaken by a team of trained technicians who met the ISAK standards for technical error of measurement^19^. Standing height was recorded to the nearest 0.5 cm using a wall mounted stadiometer. Body mass was recorded to the nearest 0.01 kg using a self-calibrating, high precision digital platform scale (MultiRange, Model ED 3300, Ebingen, Germany). WC and HC were measured twice to the nearest 0.1 cm using an inelastic retractable anthropometric tape (Lufkin W606PM Executive Diameter Tape, Lufkin, Texas, USA). If consecutive measurements were not within 2.5% of each other, a third measure was taken and the two closest measures were averaged. WC was measured at the narrowest point between the iliac crest and the 12th rib. When the technician was unable to identify the narrowest point, WC was measured at the midpoint of these landmarks. HC was measured at the widest part of the buttocks. From the anthropometric measurements, we calculated WHR (WC/HC) and WHtR = (WC/height).

### Body composition measurements

All participants received a whole-body DXA scan (GE Lunar iDXA, GE Healthcare, Milwaukee, Wisconsin, USA). Participants lay supine on the scanning table, with their body aligned within the scan plane. Radiolucent foam was placed in the axilla and between the thigh and hand to assist with post-scan segmentation. Arms were held straight by the participant’s side with palms facing towards the thigh in a semi-pronated orientation. The legs were fully extended with feet ‘shoulder-width’ apart and comfortably externally rotated. Whole-body scans were conducted using the standard (153 mm/s) or thick (80 mm/s) scan mode, as determined automatically by the scanner software using the participant’s body size. Quality assurance scans were performed each day before data collection using the manufacturer's block phantom, as well as the ‘lumbar spine’ phantom. There was no report from any operators of significant drift in calibration values throughout the study period.

All body composition outputs were batch processed and exported for statistical analysis. VAT mass was calculated using the Encore ‘CoreScan’ application (Encore v16.1, GE Lunar iDXA, GE Healthcare, Wisconsin, United States of America), wherein regions of interest for total body segmentation were inspected and adjusted by a single iDXA operator to fit the anatomical landmarks described by the manufacturer. The iDXA CoreScan software calculates the android regions of interest based on two cut-off lines placed at the top of the iliac crests and below the base of the skull^5^. The automation of these cut-off lines is often errored which can lead to significant differences in body composition outputs^20^. These cut-off lines were manually adjusted post-scan to improve the accuracy and precision of the body composition outputs. %BF, as well as android, gynoid, and VAT masses were calculated using the Encore (v16.1) software, which divides estimated soft tissue into lean and fat compartments for whole body and regional measurement of body composition. Using these body composition outputs, we calculated android-gynoid ratio (AGR = android/gynoid fat mass) and fat mass index (FMI = total fat mass/height^2^).

A subsample of 32 participants (16 men and 16 women) had two iDXA scans within the testing session. Two technicians independently adjusted the automated cut-off lines in the CoreScan software, and body composition outputs (%BF, android fat mass, gynoid fat mass, and VAT mass) were compared to assess inter- and intra-tester repeatability using an an ICC (1,1) statistical analysis.

### Statistical analysis

Participants who were more than five standard deviations away from the mean for any measure were further investigated for potential errors. All participants were stratified by sex, age (18–24.9, 25–34.9, 35–44.9, 45–54.9, 55–65 years) and BMI (< 18, 18–24.9, 25–29.9, > 30 kg/m^2^)^15^. Statistical analyses were performed in three parts using SPSS (IBM SPSS Statistics for Windows, Version 23.0. Armonk, New York, USA) and MATLAB 2015a (The MathWorks Inc., Natick, Massachusetts, USA). For the creation of female and male reference distributions, the 90th, 70th, 50th, 30th, and 10th percentiles were calculated for each sex, then plotted across the five age groups. A cubic spline interpolation (MATLAB) was used to smooth the percentile lines across age groups; the purpose of which was to enhance the clinical usability for comparison purposes.

The normality of anthropometry and body composition variables was assessed using skewness, kurtosis, and visual inspection of the histograms. Independent t-tests were performed to identify differences in normally distributed anthropometry and body composition variables between the age groups. Mann–Whitney U tests were used when one or more of the variables were not normally distributed. Using a Bonferroni correction^21^, the alpha was adjusted to 0.0063 to reduce the probability of a Type I error when making eight comparisons (four age groups by two sexes).

Correlation analysis was employed to identify significant associations between DXA-derived body composition variables. In addition, DXA-derived body composition measures were correlated with surrogate anthropometric measures of adiposity. Pearson’s correlations were performed when both variables were normally distributed; if one or more of the variables were not normally distributed, a Spearman’s rho was computed. Using a Bonferroni correction^21^, the alpha was adjusted to 0.00008 (30 correlations by two sexes). Correlation coefficients below 0.2 were considered ‘very low’, between 0.2 and 0.5 ‘low’, between 0.5 and 0.7 ‘moderate’, between 0.7 and 0.9 ‘high’, and greater than 0.9 ‘very high’^14^.

Individuals in the top 10% of VAT mass for their age and sex were considered to have high VAT mass. Specificity, sensitivity, and Youden’s Index were calculated^22,23^ for identifying these individuals with high VAT, within sex-stratified cohorts when using anthropometric measures. These values were: (1) BMI for overweight and obesity^15^ of 25.0 and 30.0 kg/m^2^, respectively; (2) WC of 80 cm for females, 94 cm for males (‘increased risk’ referred to as overweight), and 88 cm for females, 102 cm for males (‘substantially increased risk’ referred to as obese)^15^; (3) WHR of 0.85 for females and 0.90 for males^15^; (4) WHtR value of 0.50 for both females and males^7^; and (5) %BF of 37.4 for females, and 25.4% for males (‘overweight’), and 42.2% for females, and 30.0% for males (‘obese’)^24^. As the majority of the participants were White, we used the non-Hispanic White cut-off values presented by Heo and colleagues^24^. For simplicity, the thresholds for the ‘middle age’ group were used; there is minimal (≤ 2.5%) difference in the overweight and obesity cut-offs across the three age groups^24^.

## Supplementary information

Supplementary information 1

Supplementary information 2

Supplementary information 3

Supplementary information 4

Supplementary information 5

## Data Availability

Participant consent was not given for sharing of individual data. Reference normative values for DXA-derived body composition measures are available in the figures within the paper and in the supplementary materials.

## Code availability

The MATLAB (2015a) custom code used to calculate the percentile lines and create the normative distribution figures is available at: https://zenodo.org/record/4010109#.X03ldXkzaUk.
