# Supplementary material for: DXA reference values and anthropometric screening for visceral obesity in Western Australian adults
Source: Sci Rep. 2020 Oct 30;10:18731. doi: 10.1038/s41598-020-73631-x (PMC7599223; doi:10.1038/s41598-020-73631-x)
Supplement: Supplementary file 1 — Supplementary information 1 [file 41598_2020_73631_MOESM1_ESM.pdf]

DXA reference values and anthropometric screening for visceral obesity in Western Australian adults

Supplementary material 1

Jonathan M. D. Staynor, Marc K. Smith, Cyril J. Donnelly, Amar El Sallam, and Timothy R. Ackland

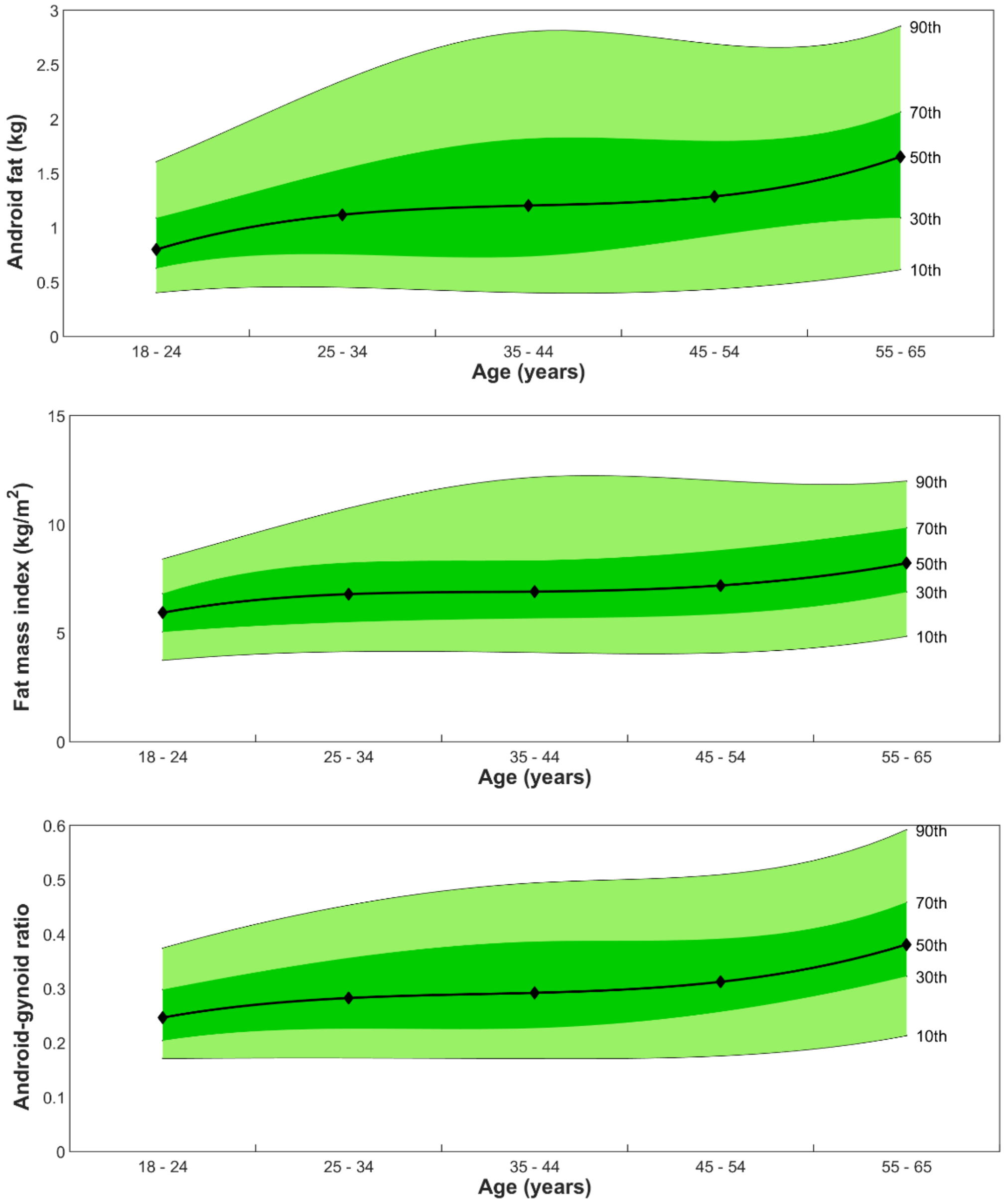

Figure S1.1: Female normative distributions across age for android fat mass (top), fat mass index (middle), and android-gynoid ratio (bottom). Solid lines, from top-bottom, represent the 90th, 70th, 50th, 30th, and 10th percentiles, respectively.

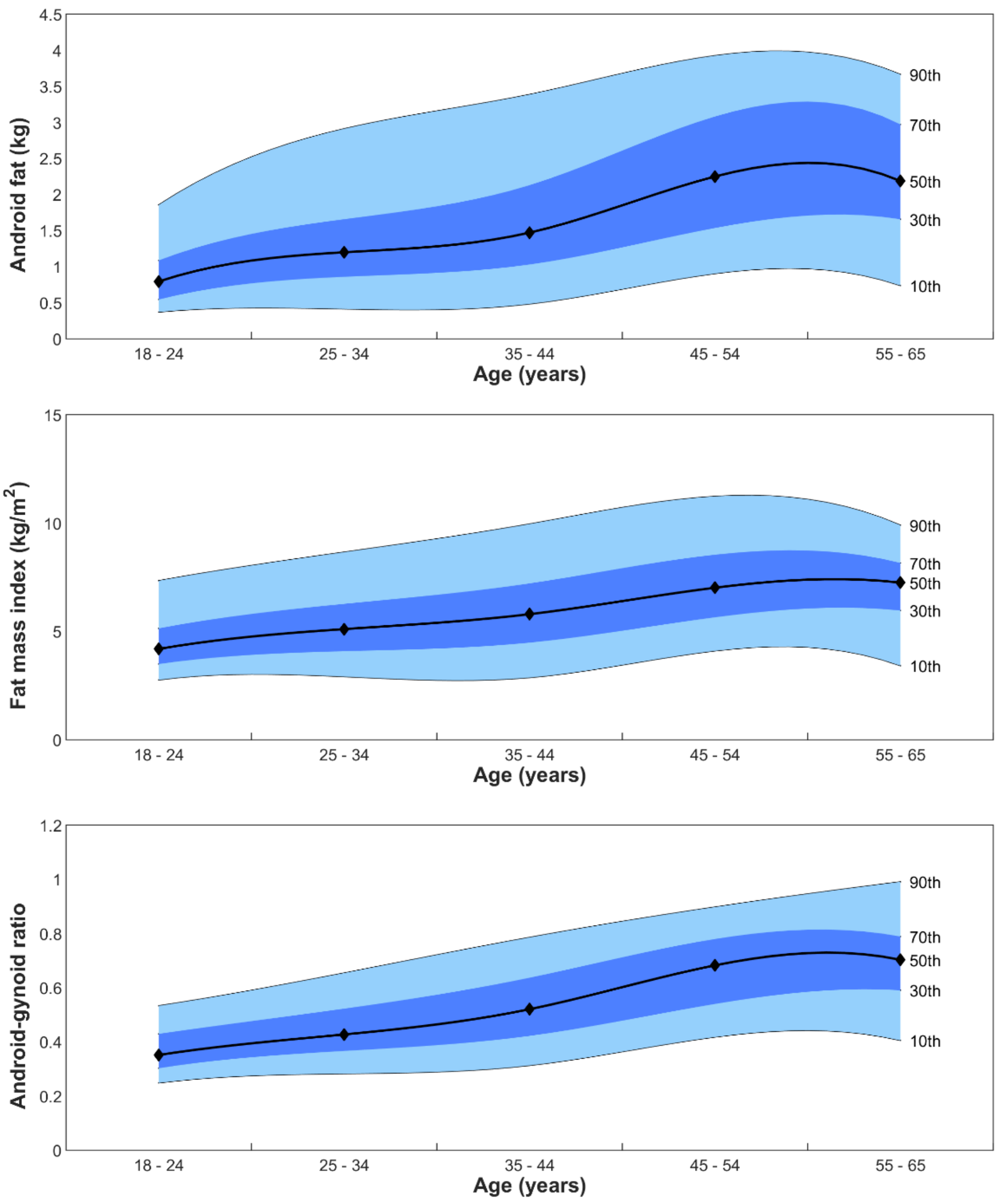

Figure S1.2: Male normative distributions across age for android fat mass (top), fat mass index (middle), and android-gynoid ratio (bottom). Solid lines, from top-bottom, represent the 90th, 70th, 50th, 30th, and 10th percentiles, respectively.

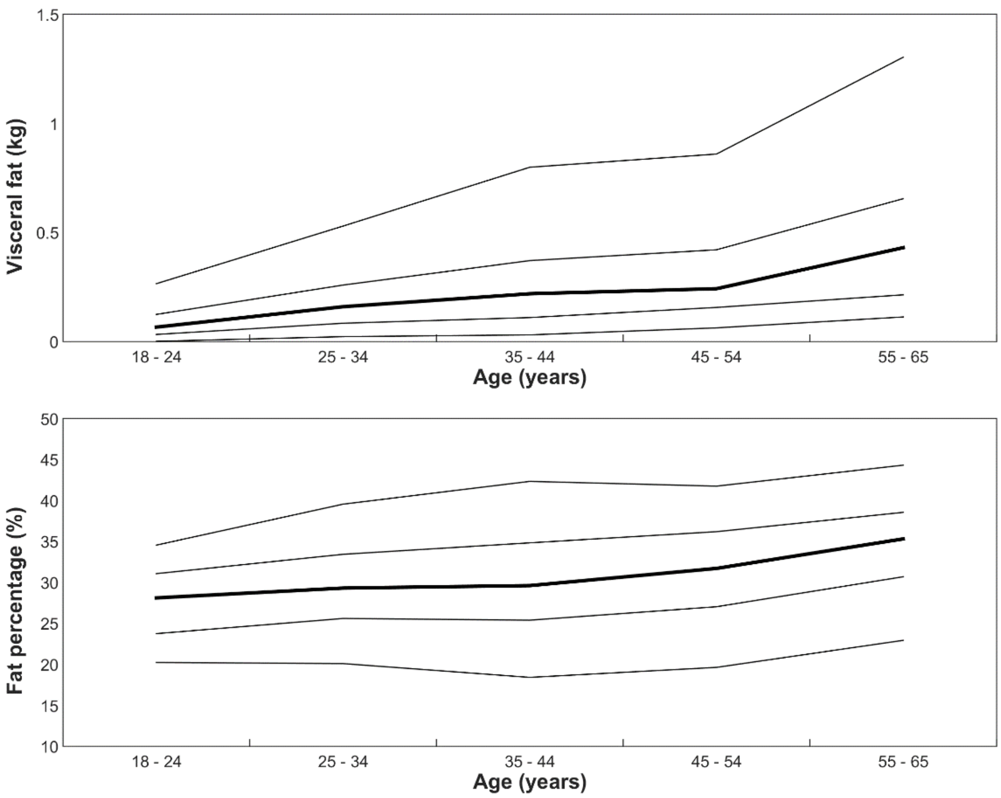

Figure S1.3: Raw (unsmoothed) female normative distributions across age for visceral fat mass (top) and fat percentage (bottom). Solid lines, from top-bottom, represent the 90th, 70th, 50th, 30th, and 10th percentiles, respectively.

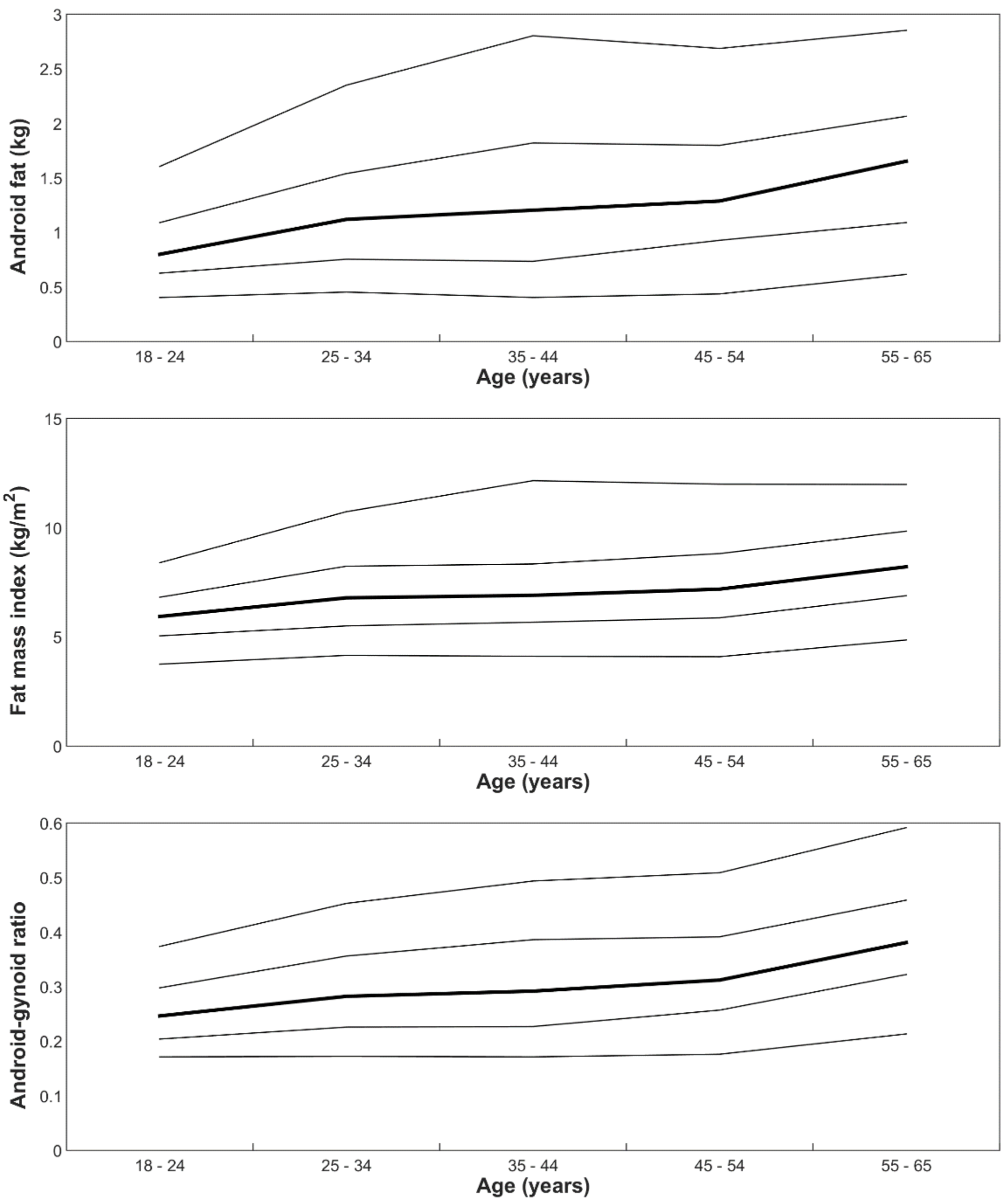

Figure S1.4: Raw (unsmoothed) female normative distributions across age for android fat (top), fat mass index (middle), and android-gynoid ratio (bottom). Solid lines, from top-bottom, represent the 90th, 70th, 50th, 30th, and 10th percentiles, respectively.

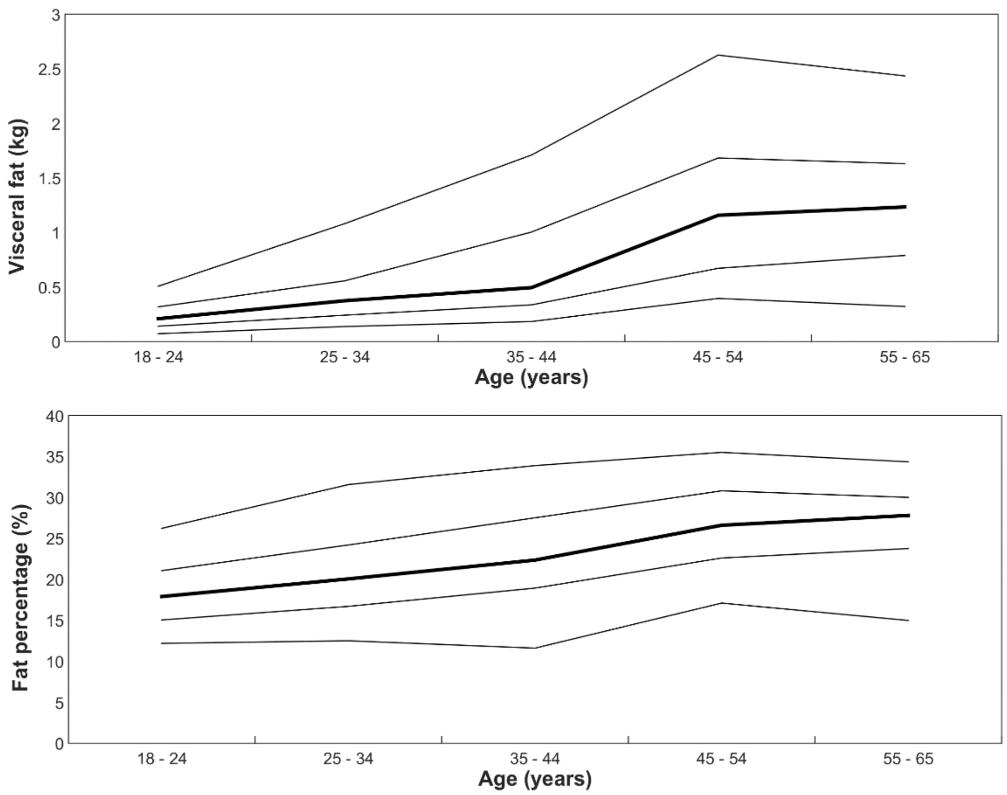

Figure S1.5: Raw (unsmoothed) male normative distributions across age for visceral fat mass (top) and fat percentage (bottom). Solid lines, from top-bottom, represent the 90th, 70th, 50th, 30th, and 10th percentiles, respectively.

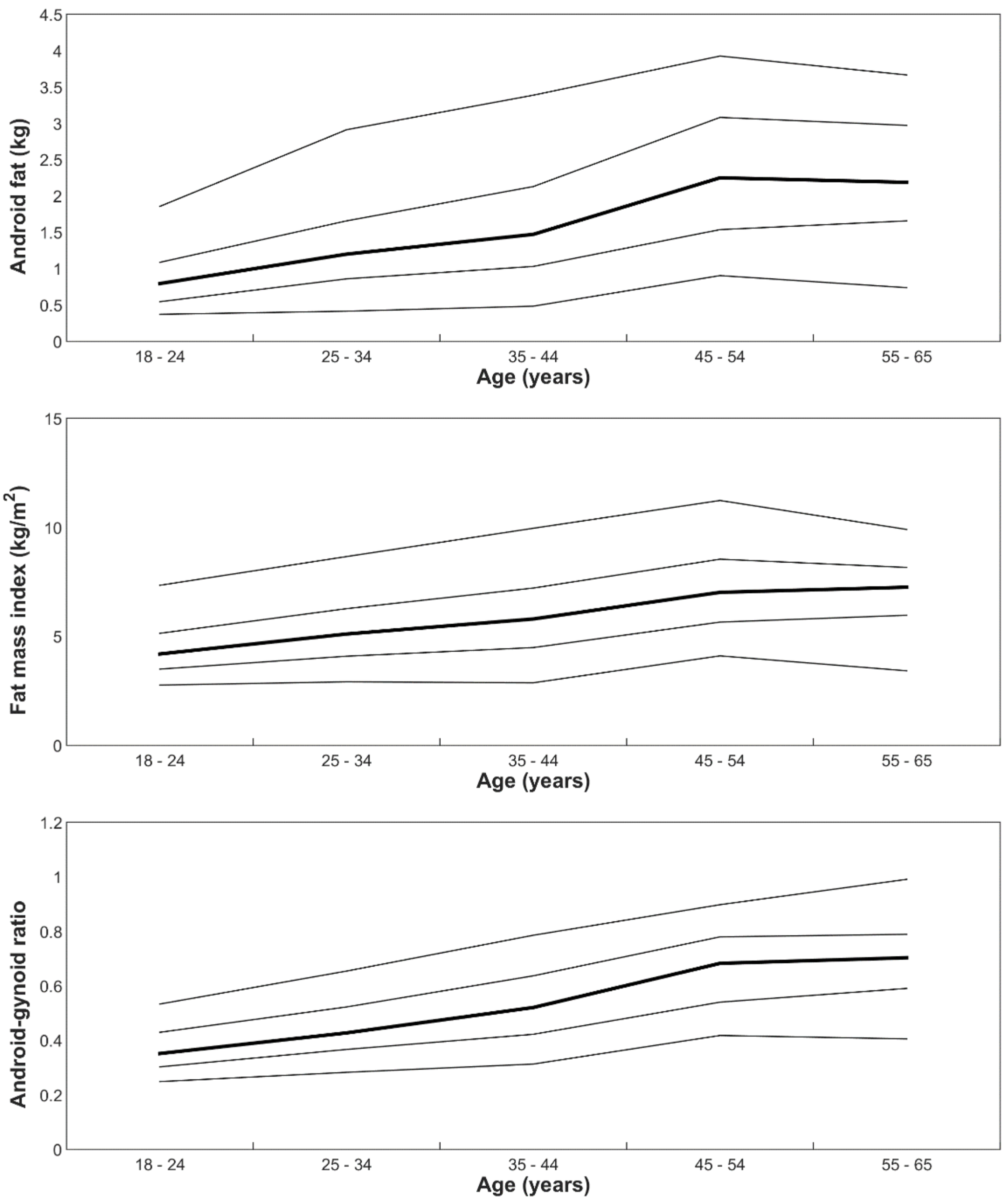

Figure S1.6: Raw (unsmoothed) male normative distributions across age for android fat (top), fat mass index (middle), and android-gynoid ratio (bottom). Solid lines, from top-bottom, represent the 90th, 70th, 50th, 30th, and 10th percentiles, respectively.
