# Supplementary material for: DXA reference values and anthropometric screening for visceral obesity in Western Australian adults
Source: Sci Rep. 2020 Oct 30;10:18731. doi: 10.1038/s41598-020-73631-x (PMC7599223; doi:10.1038/s41598-020-73631-x)
Supplement: Supplementary file 2 — Supplementary information 2 [file 41598_2020_73631_MOESM2_ESM.pdf]

DXA reference values and anthropometric screening for visceral obesity in Western Australian adults

Supplementary material 2

Jonathan M. D. Staynor, Marc K. Smith, Cyril J. Donnelly, Amar El Sallam, and Timothy R. Ackland

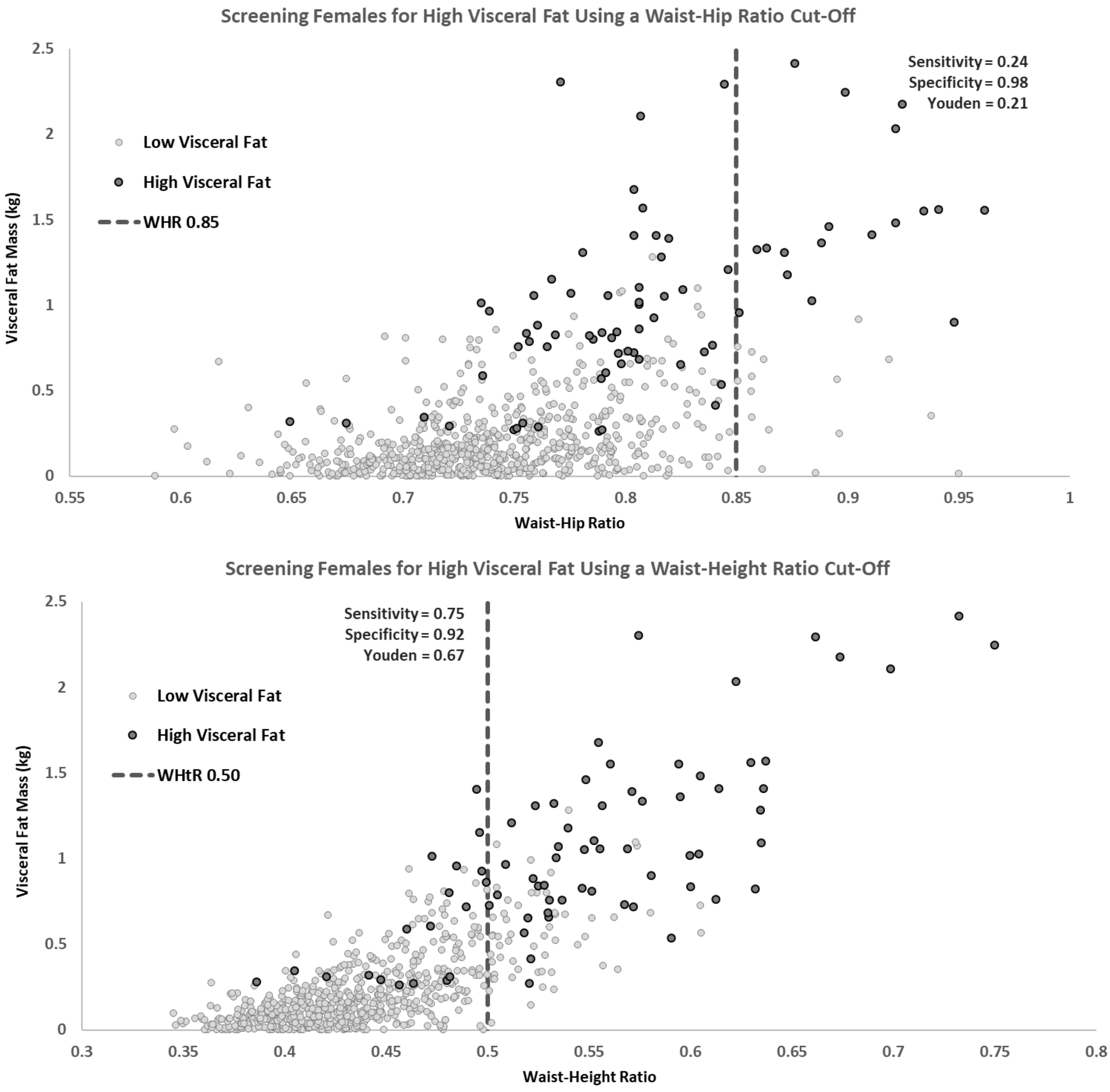

Figure S2.1: *Sensitivity, specificity and Youden's index results for identifying women with 'high VAT' (≥ 90*th *percentile for age and sex stratified VAT mass) using a WHR cut-off (top) and WHtR cut-off (bottom).*

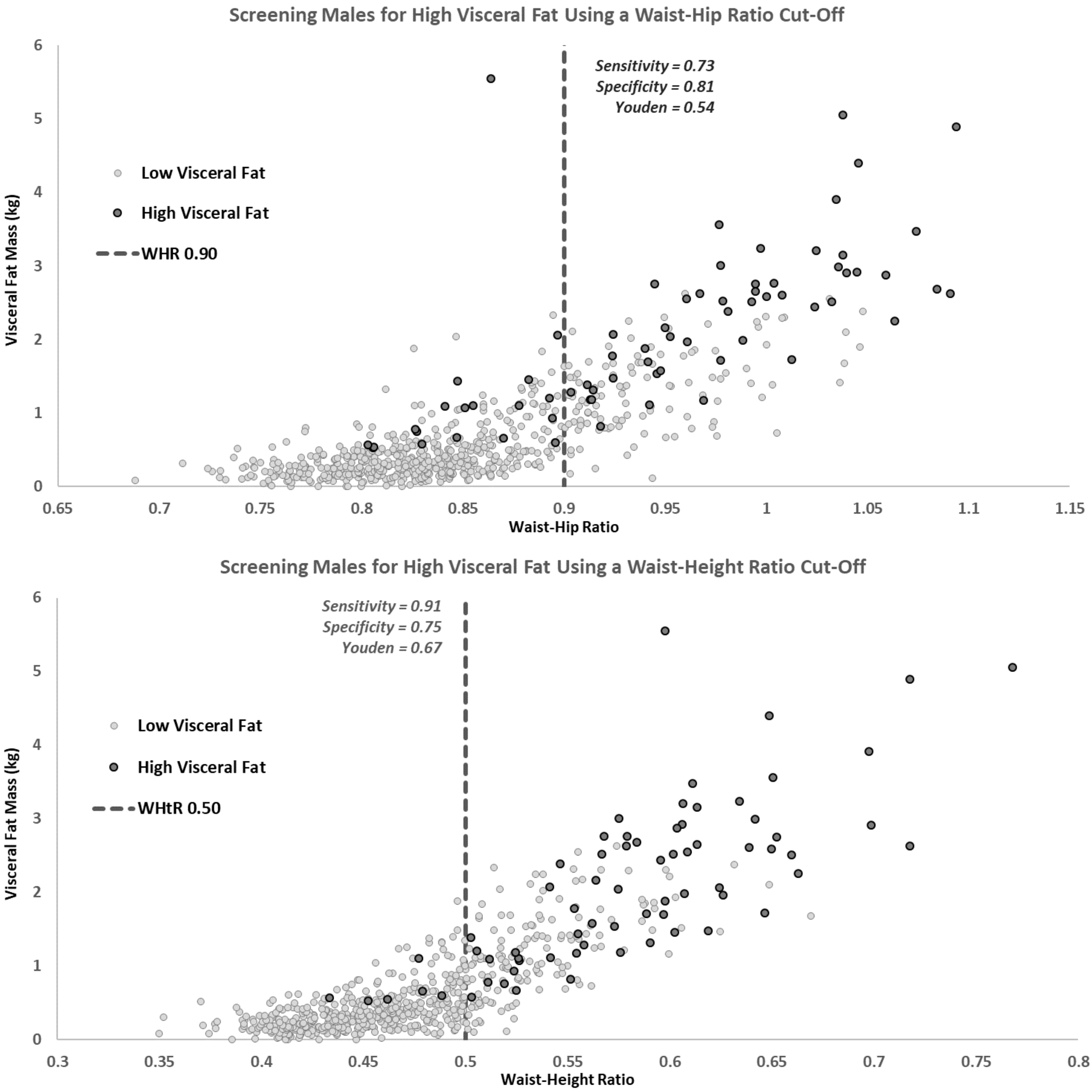

Figure S2.2: *Sensitivity, specificity and Youden's index results for identifying men with 'high VAT' (≥ 90th percentile for age and sex stratified VAT mass) using a WHR cut-off (top) and WHtR cut-off (bottom).*
