# Supplementary material for: DXA reference values and anthropometric screening for visceral obesity in Western Australian adults
Source: Sci Rep. 2020 Oct 30;10:18731. doi: 10.1038/s41598-020-73631-x (PMC7599223; doi:10.1038/s41598-020-73631-x)
Supplement: Supplementary file 3 — Supplementary information 3 [file 41598_2020_73631_MOESM3_ESM.pdf]

DXA reference values and anthropometric screening for visceral obesity in Western Australian adults

Supplementary material 3

Jonathan M. D. Staynor, Marc K. Smith, Cyril J. Donnelly, Amar El Sallam, and Timothy R. Ackland

Normality Assessment

Men

Statistics

		T_Height	T_Weight	T_BMI	WaistAve	WHR	WHtR	VATMass	FatMassAndroid	AGR	PercentFat	FMI
N	Valid	677	677	677	677	677	677	677	677	677	677	677
	Missing	0	0	0	0	0	0	0	0	0	0	0
Skewness		.144	.983	1.112	1.115	.790	1.000	2.210	1.662	.734	.437	1.307
Std. Error of Skewness		.094	.094	.094	.094	.094	.094	.094	.094	.094	.094	.094
Kurtosis		.264	2.324	3.192	1.797	.436	1.327	6.592	4.151	.052	-.350	2.764
Std. Error of Kurtosis		.188	.188	.188	.188	.188	.188	.188	.188	.188	.188	.188

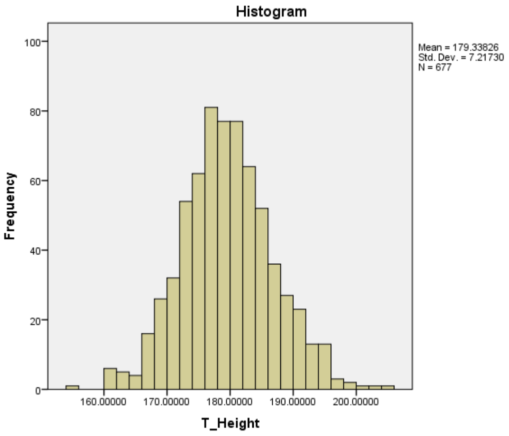

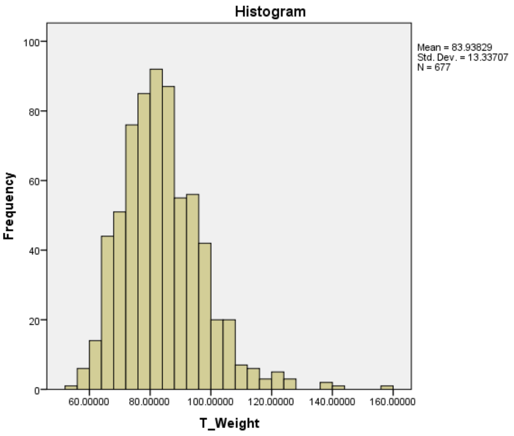

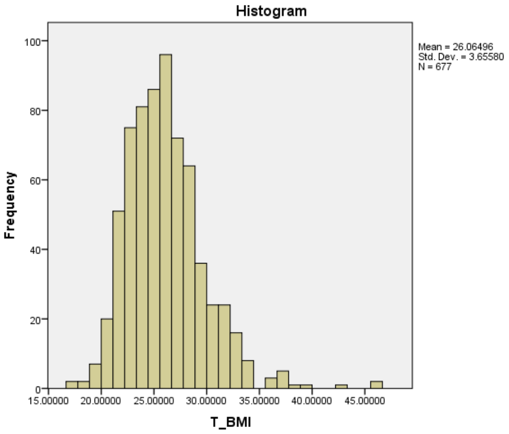

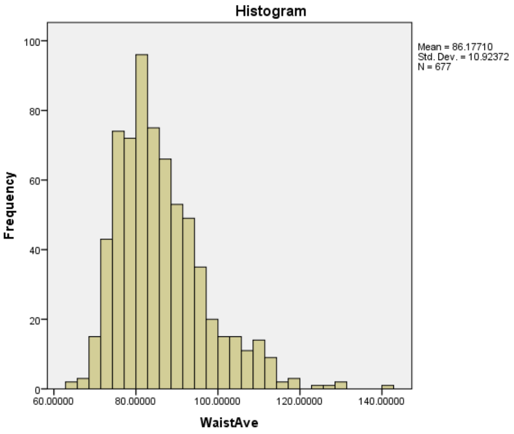

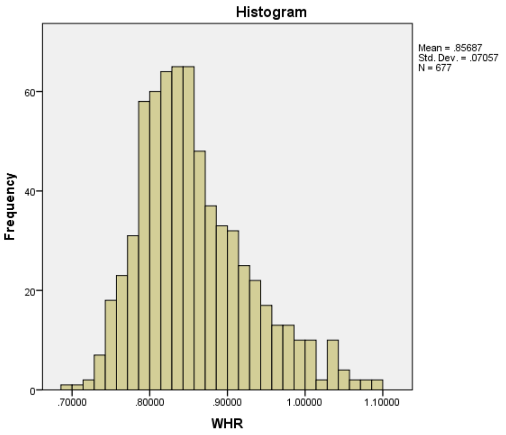

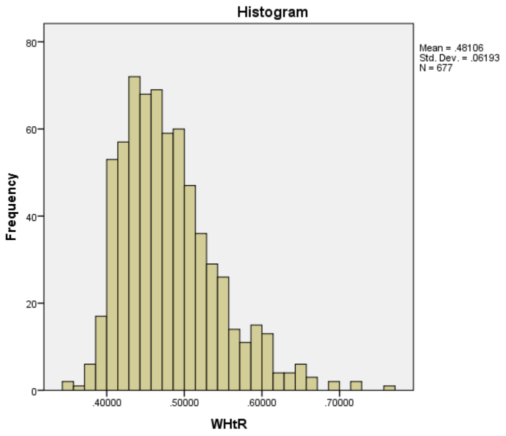

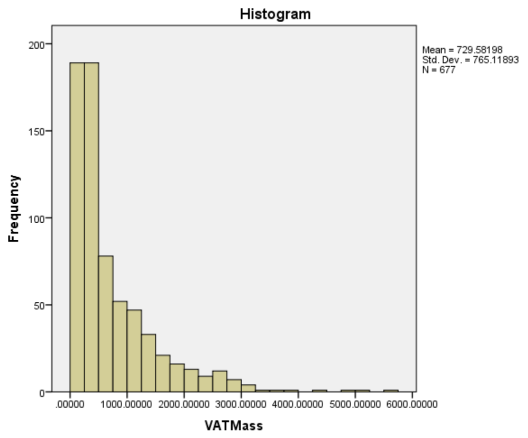
Histogram
Mean = 729.58198
Std. Dev. = 765.11893
N = 677
Frequency
VATMass

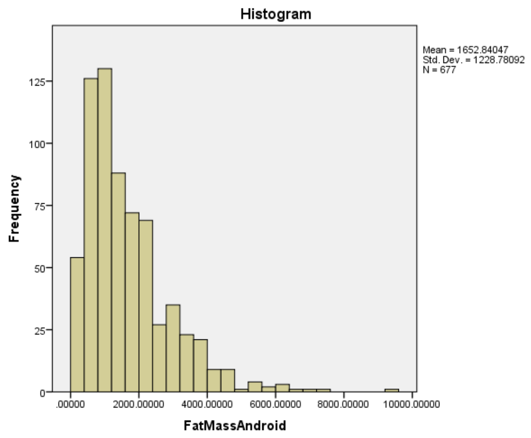
Histogram
Mean = 1652.84047
Std. Dev. = 1228.78092
N = 677
Frequency
FatMassAndroid

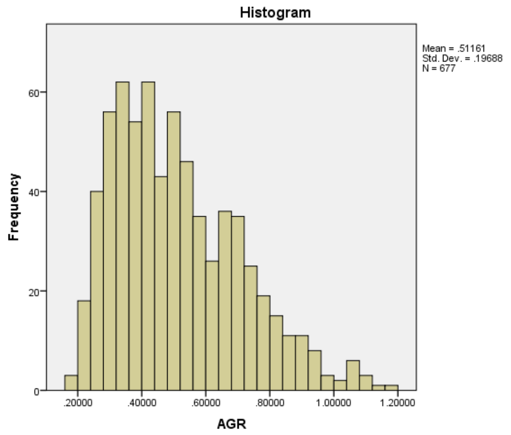
Histogram
Mean = .51161
Std. Dev. = .19688
N = 677
Frequency
AGR

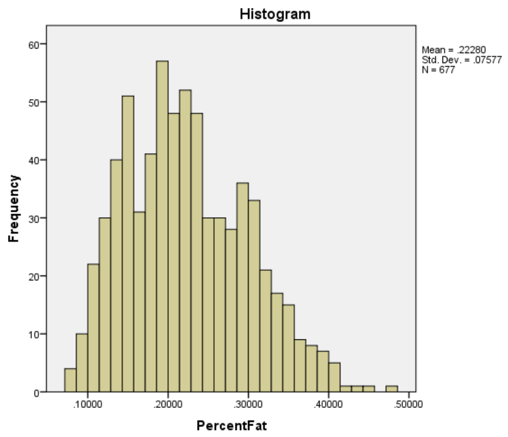
Histogram
Mean = .22280
Std. Dev. = .07577
N = 677
Frequency
PercentFat

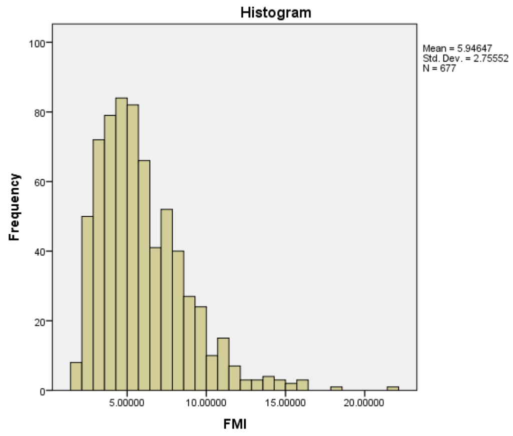
Histogram
Mean = 5.94647
Std. Dev. = 2.75552
N = 677
Frequency
FMI

Women

Statistics

		T_Height	T_Weight	T_BMI	WaistAve	WHR	WHtR	VATMass	FatMassAndroid	AGR	PercentFat	FMI
N	Valid	738	738	738	738	738	738	738	738	738	738	738
	Missing	0	0	0	0	0	0	0	0	0	0	0
Skewness		.219	1.329	1.612	1.358	.580	1.324	2.530	1.863	.917	.175	1.416
Std. Error of Skewness		.090	.090	.090	.090	.090	.090	.090	.090	.090	.090	.090
Kurtosis		-.095	3.313	4.704	2.878	.913	2.790	8.231	5.131	.853	-.348	3.045
Std. Error of Kurtosis		.180	.180	.180	.180	.180	.180	.180	.180	.180	.180	.180

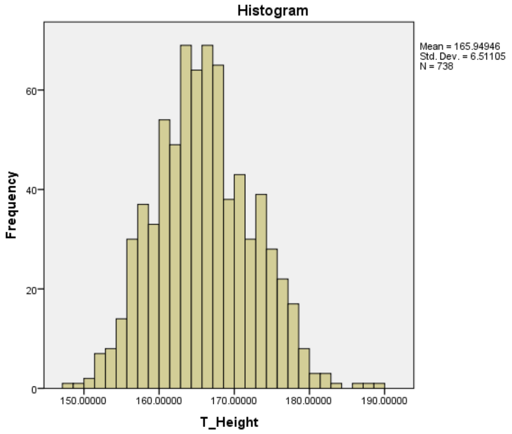

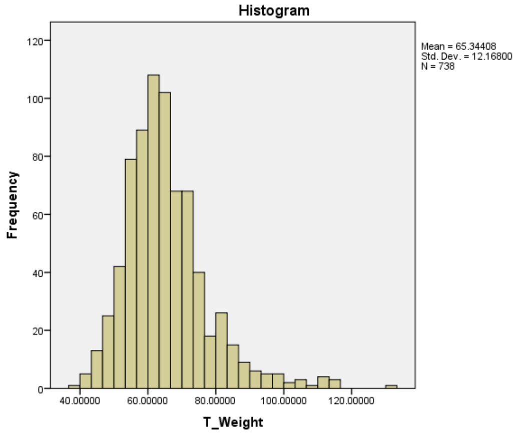

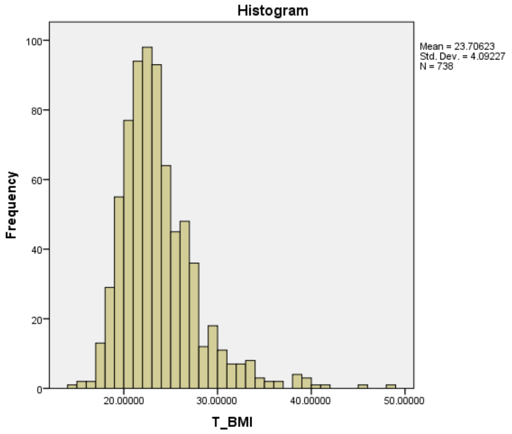

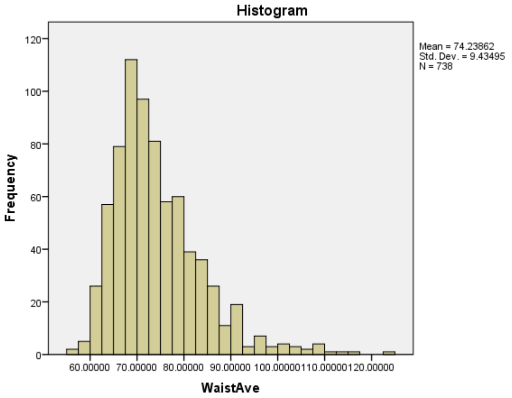

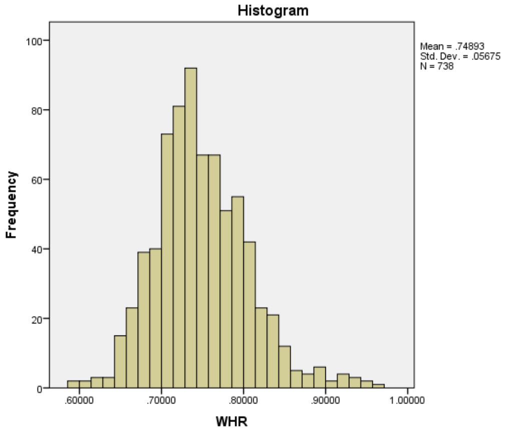

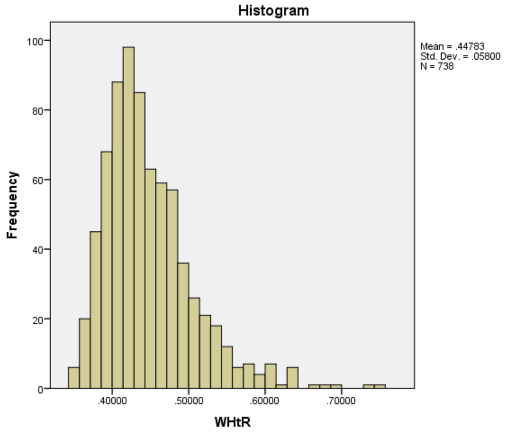

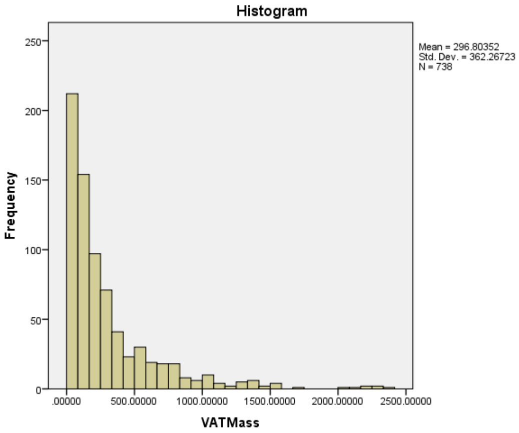
Histogram
Mean = 296.80352
Std. Dev. = 362.26723
N = 738
Frequency
VATMass

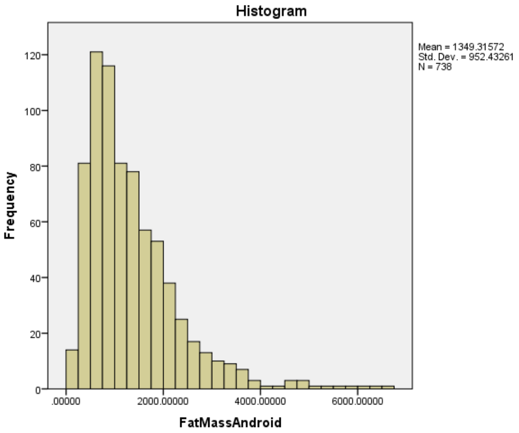
Histogram
Mean = 1349.31572
Std. Dev. = 952.43261
N = 738
Frequency
FatMassAndroid

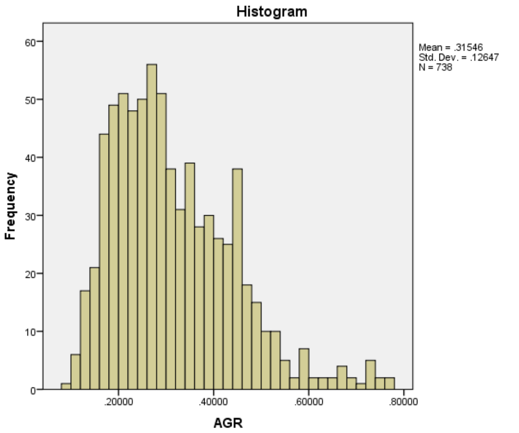
Histogram
Mean = .31546
Std. Dev. = .12647
N = 738
Frequency
AGR

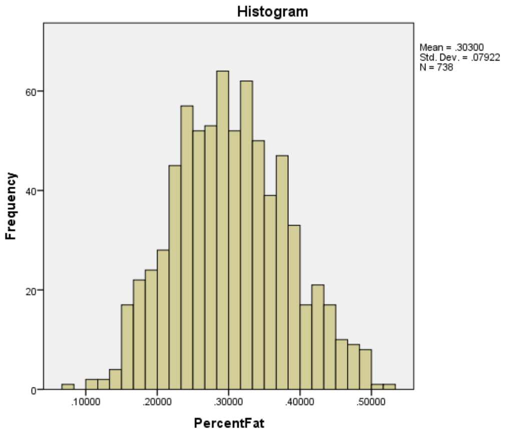
Histogram
Mean = .30300
Std. Dev. = .07922
N = 738
Frequency
PercentFat

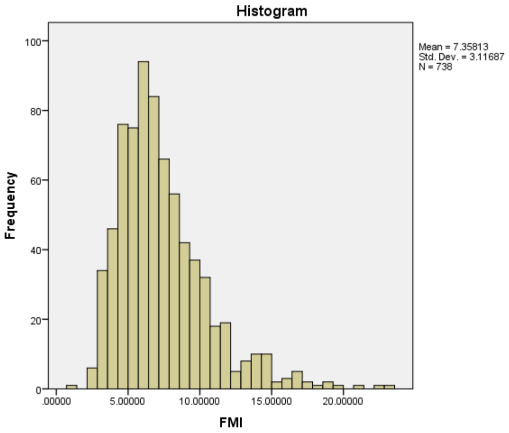
Histogram
Mean = 7.35813
Std. Dev. = 3.11687
N = 738
Frequency
FMI
