# Supplementary material for: DXA reference values and anthropometric screening for visceral obesity in Western Australian adults
Source: Sci Rep. 2020 Oct 30;10:18731. doi: 10.1038/s41598-020-73631-x (PMC7599223; doi:10.1038/s41598-020-73631-x)
Supplement: Supplementary file 4 — Supplementary information 4 [file 41598_2020_73631_MOESM4_ESM.pdf]

DXA reference values and anthropometric screening for visceral obesity in Western Australian adults

Supplementary material 4

Jonathan M. D. Staynor, Marc K. Smith, Cyril J. Donnelly, Amar El Sallam, and Timothy R. Ackland

Table 1. Anthropometric and DXA-derived body composition variables by sex and age group in participants whose self-reported ethnicity was 'white'.

	Age (years)					
	All (18-65)	18-24	25-34	35-44	45-54	55-65
Females						
n	616	119	166	109	140	82
Height (cm)	166.9 ± 6.3	169.0 ± 6.2	167.6 ± 6.2	167.0 ± 5.9	166.0 ± 6.1	**163.7 ± 5.8***
Weight (kg)	64.1 (58.5-71.7)	62.8 (56.9-68.0)	65.2 (59.9-72.9)	65.3 (58.9-71.5)	63.7 (57.9-72.8)	64.8 (58.7-72.7)
Body mass index (kg/m^2)	23.1 (21.2-25.7)	22.0 (20.6-23.4)	**23.4 (21.4-26.1)***	23.1 (21.3-25.8)	23.2 (21.5-26.2)	24.1 (21.8-27.1)
Waist circumference (cm)	72.6 (68.1-79.5)	68.8 (65.7-72.5)	**72.0 (68.3-78.2)***	73.9 (68.5-79.7)	74.8 (69.6-82.1)	76.7 (70.5-84.5)
Waist-Hip ratio	0.75 ± 0.06	0.72 ± 0.04	0.74 ± 0.05	0.75 ± 0.05	0.76 ± 0.06	0.77 ± 0.07
Waist-height ratio	0.44 (0.41-0.48)	0.41 (0.39-0.43)	**0.43 (0.41-0.47)***	0.45 (0.41-0.48)	0.45 (0.42-0.49)	0.47 (0.43-0.51)
Visceral adipose tissue (kg)	0.17 (0.06-0.37)	0.05 (0.02-0.13)	**0.14 (0.06-0.30)***	0.20 (0.79-0.40)	0.24 (0.13-0.50)	**0.43 (0.16-0.80)***
Abdominal fat (kg)	1.09 (0.67-1.79)	0.80 (0.57-1.18)	**1.02 (0.67-1.70)***	1.05 (0.62-1.85)	1.28 (0.76-2.02)	1.65 (0.96-2.36)
Android-gynoid fat ratio	0.31 ± 0.13	0.25 ± 0.08	**0.29 ± 0.11***	0.31 ± 0.13	0.33 ± 0.13	**0.39 ± 0.15***
Percentage body fat (%)	30.0 ± 8.1	27.2 ± 6.5	29.6 ± 8.1	29.3 ± 8.5	31.1 ± 8.0	34.1 ± 8.2
Fat mass index (kg/m^2)	6.7 (5.2-8.8)	5.8 (4.9-7.0)	6.7 (4.9-8.8)	6.4 (4.9-8.4)	7.2 (5.6-94)	8.2 (5.9-10.2)
Males						
n	549	104	171	120	81	73
Height (cm)	180.5 ± 6.9	181.9 ± 7.6	181.5 ± 6.6	179.9 ± 7.1	179.3 ± 5.6	178.5 ± 6.7
Weight (kg)	84.4 (76.2-93.0)	80.0 (72.2-87.6)	**84.9 (77.5-91.9)***	84.4 (76.4-94.1)	87.3 (79.9-96.8)	84.6 (76.4-96.0)
Body mass index (kg/m^2)	26.0 (23.6-28.1)	24.0 (22.2-26.4)	**26.0 (23.5-27.9)***	26.1 (24.2-28.5)	27.4 (25.5-29.6)	27.0 (25.1-29.4)
Waist circumference (cm)	85.1 (79.3-93.0)	77.9 (75.0-82.8)	**83.3 (79.2-89.2)***	85.9 (80.9-92.9)	**93.9 (85.1-99.8)***	93.3 (85.3-100.9)
Waist-hip ratio	0.86 ± 0.07	0.81 ± 0.04	**0.83 ± 0.05***	**0.86 ± 0.06***	**0.91 ± 0.07***	0.93 ± 0.07
Waist-height ratio	0.47 (0.44-0.52)	0.43 (0.41-0.46)	**0.46 (0.43-0.49)***	**0.48 (0.45-0.51)***	**0.52 (0.47-0.56)***	0.53 (0.48-0.56)
Visceral adipose tissue (kg)	0.48 (0.25-1.09)	0.21 (0.12-0.36)	**0.39 (0.23-0.65)***	**0.52 (0.31-1.09)***	**1.22 (0.56-1.87)***	1.28 (0.66-1.89)
Abdominal fat (kg)	1.33 (0.74-2.23)	0.70 (0.46-1.06)	**1.20 (0.74-1.88)***	1.52 (0.80-2.25)	**2.27 (1.34-3.31)***	2.20 (1.63-3.13)
Android-gynoid fat ratio	0.51 ± 0.20	0.35 ± 0.11	**0.46 ± 0.15***	**0.53 ± 0.18***	**0.67 ± 0.19***	0.68 ± 0.21
Percentage body fat (%)	21.5 ± 7.8	17.6 ± 5.3	**21.0 ± 0.07***	22.6 ± 8.3	**26.3 ± 7.1***	26.4 ± 7.0
Fat mass index (kg/m^2)	5.4 (3.8-7.6)	4.0 (3.1-5.1)	**5.0 (3.8-6.8)***	5.7 (4.1-7.7)	**7.3 (5.1-9.1)***	7.3 (5.6-8.9)

Study cohort characteristics by sex and age. Normally distributed variables represented by mean ± standard deviation. Non-normally distributed variables represented with median (25th percentile – 75th percentile). A bold ***** denotes $p < 0.0063$ versus preceding age group.
